# Refget: standardized access to reference sequences

**DOI:** 10.1093/bioinformatics/btab524

**Published:** 2021-07-14

**Authors:** Andrew D Yates, Jeremy Adams, Somesh Chaturvedi, Robert M Davies, Matthew Laird, Rasko Leinonen, Rishi Nag, Nathan C Sheffield, Oliver Hofmann, Thomas M Keane

**Affiliations:** European Molecular Biology Laboratory, European Bioinformatics Institute, Wellcome Genome Campus, Hinxton, Cambridge CB10 1SD, UK; Global Alliance for Genomics and Health; Global Alliance for Genomics and Health; Ontario Institute for Cancer Research, Toronto, ON, Canada; European Molecular Biology Laboratory, European Bioinformatics Institute, Wellcome Genome Campus, Hinxton, Cambridge CB10 1SD, UK; Google Summer of Code; Global Alliance for Genomics and Health; Wellcome Sanger Institute, Wellcome Genome Campus, Hinxton CB10 1SA, UK; European Molecular Biology Laboratory, European Bioinformatics Institute, Wellcome Genome Campus, Hinxton, Cambridge CB10 1SD, UK; European Molecular Biology Laboratory, European Bioinformatics Institute, Wellcome Genome Campus, Hinxton, Cambridge CB10 1SD, UK; European Molecular Biology Laboratory, European Bioinformatics Institute, Wellcome Genome Campus, Hinxton, Cambridge CB10 1SD, UK; Global Alliance for Genomics and Health; Center for Public Health Genomics, School of Medicine, University of Virginia, Charlottesville, VA 22903, USA; Global Alliance for Genomics and Health; University of Melbourne Centre for Cancer Research, University of Melbourne, Melbourne, VIC, Australia; European Molecular Biology Laboratory, European Bioinformatics Institute, Wellcome Genome Campus, Hinxton, Cambridge CB10 1SD, UK; Global Alliance for Genomics and Health

## Abstract

**Motivation:**

Reference sequences are essential in creating a baseline of knowledge for many common bioinformatics methods, especially those using genomic sequencing.

**Results:**

We have created refget, a Global Alliance for Genomics and Health API specification to access reference sequences and sub-sequences using an identifier derived from the sequence itself. We present four reference implementations across in-house and cloud infrastructure, a compliance suite and a web report used to ensure specification conformity across implementations.

**Availability and implementation:**

The refget specification can be found at: https://w3id.org/ga4gh/refget.

**Supplementary information:**

Supplementary data are available at *Bioinformatics* online.

## 1 Introduction

Reference genome sequences are central to genomic interpretation and to defining a baseline of knowledge upon which our understanding of biological systems, phenotypes and variation are based. The ability to interpret such data is essential for the delivery of genomics into the clinic. As precision medicine becomes mainstream in healthcare systems, organizations such as the Global Alliance for Genomics and Health (GA4GH) are developing interoperable standards to ensure the discovery and provenance of baseline knowledge.

Reference sequences suffer from two issues: sequence identity and non-standardized access. Genomic analysis, such as read alignment, typically use a FASTA-formatted collection of sequences downloaded from a provider with inconsistent naming, e.g. INSDC (Karsch-Mizrachi *et al.*, 2018), Ensembl (Yates *et al.*, 2020) or UCSC (Lee *et al.*, 2020). For example, chromosome 1 from GRCh38 (GCA_000001405.15) is known as chr1 from hg38 (UCSC), 1 from GRCh38 (Ensembl) or CM000663.2 (INSDC). When it is critical to unambiguously identify an underlying reference sequence, it is better to use an identifier derived from the sequence itself, such as a cryptographic checksum digest. This method is employed in the CRAM format (Hsi-Yang Fritz *et al.*, 2011), which uses a MD5 digest to identify the correct reference during read reconstitution. The European Nucleotide Archive (ENA) developed the CRAM reference registry (CRR) to retrieve reference sequences by an MD5 sequence checksums. Similar ideas have been employed by tximeta to aid reproducible RNA-seq analysis (Love *et al.*, 2020).

This manuscript describes a new application programming interface (API), called refget, which enables retrieval of full-length sequences or sub-sequences via a checksum identifier, returns metadata associated with an identifier and maintains compatibility with the CRR. Our API operates over HTTP(s) and so is accessible in all main-stream programming languages. We also present four implementations of the refget specification deployed across in-house and cloud infra-structures, and a toolkit to assess implementation compliance.

## 2 Results

The refget protocol operates with a client providing a supported digest identifier with an optional linear or circular genomic coordinate range, specified as URL parameters or a Range header, via a HTTP(s) GET request. An implementation responds with an unbroken stream of sequence characters. Users may request a metadata JSON document, which provides information about the sequence length, topology, known digests and any other known aliases. Finally, clients can request a JSON document of server capabilities allowing for adaptation to possible limitations of an implementation. Implementations are not restricted to a single type of reference sequence to serve and can provide DNA, mRNA, cDNA, CDS or peptide sequences. Should an implementation wish to provide a CRAM reference registry (CRR) compatible deployment they must mirror reference sequences as found in ENA.

**Fig. 1.**
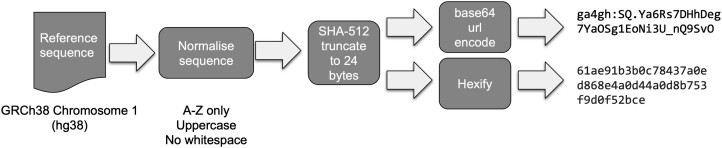
Summary of the sequence normalization and algorithm used to generate checksum identifiers for TRUNC512 and GA4GH Identifier. All methods move through the same normalization process but differ in their choice of checksum algorithm (MD5 versus SHA-512)

The Refget defines three supported identifier algorithms: MD5, TRUNC512 and GA4GH Identifier. All three algorithms normalize sequences by stripping all whitespace characters and restricting to characters in the range A-Z. We chose this as a compromise between the methods and requirements employed by CRAM, ENA and the Variation Representation Specification (VRS). MD5 is supported to maintain compatibility with CRAM format. However, hash collisions are a known weakness and to mitigate this concern, we have used the sha512t24u identifier scheme (Hart and Prlić, 2020) (also known as the GA4GH identifier) as employed by the VRS standard. In addition, we created a parallel format called TRUNC512, which represents sha512t24 as a hex string to maintain a similar representation to MD5. These schemes are described in Figure 1. However, sha512t24u is the preferred representation due to its use in VRS. We tested sha512t24u to the MGnify (Mitchell *et al.*, 2018) May 2019 protein database of ∼1 billion entries and found no collisions (see Supplementary Material). To retrieve a reference sequence, a client constructs a URL such as https://www.ebi.ac.uk/ena/cram/sequence/3332ed720ac7eaa9b3655c06f6b9e196, sets the acceptable media type to text/plain and uses a HTTP library such as Python’s requests package to negotiate the request (see Supplementary Material for additional examples).

## 3 Implementation and compliance

Four implementations exist across a diverse range of providers including ENA, Amazon Web Services (AWS) and Heroku (see Supplementary Materials). We developed a refget compliance documentation (https://compliancedoc.readthedocs.io/) and library suite (https://pypi.org/project/refget-compliance/) to ensure implementation compatibility. The compliance toolkit mandates an implementation hosts three sequences; Enterobacteria phage phiX174 sensu lato (NC_001422.1) and Saccharomyces cerevisiae S288C chromosomes I (BK006935.2) and IV (BK006938.2). Certain tests can be skipped if a pass was not possible e.g. we do not test circular sequence retrieval if a server declares it does not support circular sequences. Tests are run daily against all known implementations and a report is published at https://w3id.org/ga4gh/refget/compliance. We have also implemented a local Python interface in the refget Python package, hosted at PyPI (https://pypi.org/project/refget/). This package provides a local implementation of the refget protocol with SQLite, or MongoDB back-ends, and can connect to a remote API to provide local caching of retrieved results to improve performance for applications that require repeated lookups. It also provides Python functions to compute refget identifiers from within Python using raw sequences or FASTA files. Use of this package is shown below.

import refget

srv = “https://w3id.org/”

url = srv + “ga4gh/refget/reference/sequence/”

rgc = refget. RefGetClient(url)

rgc.refget(“6681ac2f62509cfc220d78751b8dc524”, start = 0, end = 10)

## 4 Discussion

Reference sequences are fundamental for providing a stable method of describing genomic variation and annotation. Refget formalzses a method for generating identifiers from reference sequence and specifies an API to retrieve sequences, sub-sequences and metadata. The specification is easy to implement with a mechanism to assert specification compliance. Refget can host any type of reference sequence, allows deployments to implement subsets of functionalities and provides a mechanism for deployments to programmatically declare this. Future work includes the definition of a reference sequence collection using checksums and sequence metadata, e.g. a genome and to provide a way to convert between known reference sequence names to refget identifiers.

## Supplementary Material

btab524_Supplementary_Data
